# Control of high-speed jumps: removing rotation from the jumps of locusts (*Schistocerca gregaria*)

**DOI:** 10.1242/jeb.249846

**Published:** 2026-08-07

**Authors:** Chloe K. Goode, Scott Dixon, Gregory P. Sutton

**Affiliations:** Department of Biology, University of Lincoln, Joseph Banks Laboratories, Beevor Street, Lincoln LN6 7DL, UK

**Keywords:** LaMSA, Pitch, Locust, Energetics, Biomechanics, Jumping

## Abstract

To jump, locusts (*Schistocerca gregaria*) use a latch-mediated spring-actuated (LaMSA) mechanism in their metathoracic legs, which gradually stores and then rapidly releases elastic energy, propelling them into the air. This system consistently results in a head-up, tail-down pitch of the body immediately after take-off, caused by a thus far unknown underlying mechanism. In this study, we aimed to test the hypothesis that head-up, tail-down angular rotation is a product of the locust's centre of mass being positioned off-centre to the force vector produced by the metathoracic legs; therefore, shifting the centre of mass forwards should counterbalance any torque acting around it. This was achieved experimentally by attaching a small mass, ranging from 3.4 to 13.6% of the locust's body mass, to the head of a locust before filming it jump to a target substrate. The resultant angular velocity from these jumps decreased as the added mass increased, with a mass equivalent to 10.2% of the body mass resulting in the complete elimination of angular velocity and therefore an entirely linear jump. When added mass totalled 13.6%, negative angular velocities were recorded (head-down, tail-up). This was matched with a corresponding decrease in the rotational energy percentage of each jump's total energy budget, and a slight decrease in linear velocity for the largest added mass. These findings strongly support the theory that locusts do not independently control their body pitch at take-off, and that changes to the body mass distribution can counter it.

## Teaser

**Summary:** Moving the position of the centre of mass with a counter balance reduces the angular rotation of locust (*Schistocerca gregaria*) jumps.

## INTRODUCTION

Locusts (*Schistocerca gregaria*), like fleas and froghoppers, jump using a latch-mediated spring-actuated (LaMSA) system, allowing them a rapid means of locomotion with a linear velocity that is independent of body size (Brown, 1967; Heitler, 1974; Goode and Sutton, 2023). Prior to take-off, the metathoracic legs of the locust are flexed and latched into position by contraction of the flexor tibiae muscle, which extends ventrally along the femur. Next, slow contraction of the extensor tibiae, the largest muscle in the femur, deforms two spring-like structures – the cuticular semi-lunar process and the extensor apodeme – which store elastic potential energy. Relaxation of the flexor tibiae triggers the release of the spring-like structures, which recoil, rapidly converting the stored elastic energy into kinetic energy, forcefully extending the metathoracic leg and propelling the locust into the air (Heitler, 1974, 1977; Bennet-Clark, 1975; Cofer et al., 2010).

Successful jumps to a target substrate require the correct control of speed (Sobel, 1990), a product of the amount of elastic energy stored in the leg (Bennet-Clark, 1975), and an appropriate trajectory, determined by postural adjustments that occur prior to and during take-off (Santer et al., 2005; Sutton and Burrows, 2008; Gvirsman et al., 2016). The position of the prothoracic and mesothoracic legs determines the azimuth of the jump (Santer et al., 2005; Gvirsman et al., 2016). The angle of elevation is predominantly influenced by the relative positioning of the metathoracic leg's femur and coxa (Sutton and Burrows, 2008; Gvirsman et al., 2016). Jumping locusts also exhibit rotational movements around all three body axes during take-off. For example, substantial pitch rotations have been observed in various studies (Pond, 1972; Cofer et al., 2010; Gvirsman et al., 2016). However, the extent to which a locust can independently control this angular velocity is less certain compared with that of other insects. For example, praying mantises can control their body's pitch angle by counter rotation of the legs and abdomen (Burrows et al., 2015; Sutton et al., 2016); however, the prothoracic legs of locusts are of too little mass to allow substantial benefit from the same manner of adjustment. Locust body pitch, after take-off, is predominantly governed by a positive torque produced by the flexion of the dorsal longitudinal muscles (Baader, 1990; Cofer et al., 2010), consistently resulting in a head-up, tail-down pitch during jumping (Bennet-Clark, 1975; Sutton and Burrows, 2008; Cofer et al., 2010; Gvirsman et al., 2016). However, this muscle-generated counter torque only occurs once the locust is airborne (Baader, 1990) and therefore cannot influence pitch during the initial take-off phase of a jump.

Our previous study (Goode and Sutton, 2023) found that on average, locusts dedicate 1.3% of a jump's total kinetic energy budget to angular rotation, with the remaining 98.7% attributed to linear translation. Rotational kinetic energy increased proportionally and linearly with translational kinetic energy, further indicating that locusts do not independently control rotational and translational velocity during the initial take-off phase of a jump, leading us to believe that the mechanism generating angular rotation is a by-product. This could be achieved through a geometrical imbalance of the mass distribution of the locust, whereby the locust's centre of mass is positioned a fixed distance away from the force vector generated by the metathoracic legs during take-off. The distance between the centre of mass and force vector would act as a moment arm, generating torque around the centre of mass, subsequently producing angular rotation of the body during take-off. If the assumption that locusts do not independently control rotation is true, then the size of such a moment arm could be manipulated by artificially shifting the locusts’ centre of mass to increase or decrease spinning during a jump.

In this study, we aimed to test a quantitative hypothesis that relates angular velocity to the location of the locust's centre of mass. This was achieved by adding masses of known quantities to the locust's head, with the intention of bringing the centre of mass forward and subsequently putting the centre of mass in line with the force vector from the legs, thus balancing the rotational torques on the body. If, as hypothesised, all angular rotation is produced by leg forces acting off-centre to the centre of mass, then there should be a value of mass which, when added to the head of the locust, entirely removes rotation, producing a completely linear translating jump. A more comprehensive understanding of how locusts control their angular velocity is crucial for those designing miniature jumping robots, which must execute rapid and accurate jumps under component-limiting size constraints (Chen et al., 2011; Nguyen and Park, 2012; Zaitsev et al., 2015).

## MATERIALS AND METHODS

### Model

**Fig. 1.**
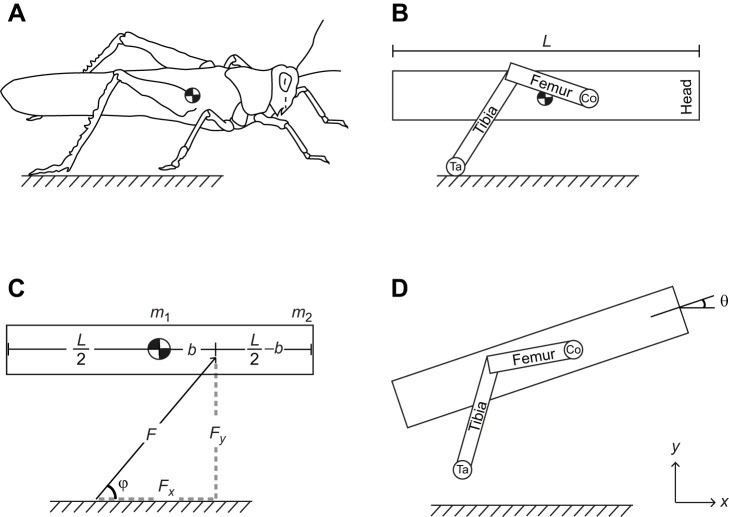
**Locust model.** (A) The locust at rest. The centre of mass (COM) is positioned posterior to the coxa of the metathoracic leg. (B) Model of the locust body as a uniform rod, length *L*. The metathoracic femur attaches to the body at the coxa (Co) joint. The tibia is connected to the tarsi (Ta), which, prior to take-off, is in contact with the ground. (C) Force model shows the force (*F*) from the metathoracic legs, split into its horizontal (*F_x_*) and vertical (*F_y_*) components, acting on the locust body at a known constant angle phi (ϕ), from an unknown distance (*b*) from the COM. Body mass (*m*_1_) is initially coincident with the COM, until the hat mass (*m*_2_) is added to the head of the locust, causing the COM to shift forward. (D) At take-off, the metathoracic tarsi leave the ground. Body angle (θ) is measured relative to ground.

To determine the exact amount of mass necessary to remove angular rotation from the locust's jump, we first produced a simple model of the locust (Fig. 1B). In this model, the body of the locust was modelled as a uniform beam with a centre of mass representing the total mass of the animal. Two additional light and inextensible beams were used to represent the femur and tibia of the metathoracic hind legs, which produce all of the jump force. In this model, there is one point of contact with the ground, labelled as the position of the Tarsi. The force (*F*) generated by the metathoracic legs was applied to the body a distance (*b*) away from the centre of mass, at a constant angle (Sutton and Burrows, 2008) phi (ϕ)=45 deg (Queathem and Full, 1995) from the ground (Fig. 1C). All of the following calculations for the kinematics and energetics of the locust were made using the assumptions of this basic model and the equations of motion, for which the mathematical constants are shown in Table 1.

**Table 1.** Mathematical constants

Constant	Symbol	Units
Body mass	*m*_1_	g
Hat mass	*m*_2_	g
Hat mass percentage	*h*%	%
Total mass	*m*	g
Body length	*L*	mm
Moment arm length	*b*	mm
Leg force	*F*	N
*y* direction force	*F_y_*	N
*x* direction force	*F_x_*	N
Linear velocity	*v*	m s^−1^
Angular velocity	$\dot{\theta }$	rad s^−1^
Angular acceleration	$\ddot{\theta }\;$	rad s^−2^
Body angle	θ	rad
Force angle phi	ϕ	deg

#### Equations of motion

Translational kinetic energy was defined as:

(1) $$KE_{T}=\frac{1}{2}mv^{2},$$

where *m* is mass and *v* is linear velocity.

Rotational kinetic energy was defined as:

(2) $$KE_{R}=\frac{1}{2}I{\dot{\theta }}^{2},$$

where *I* is the moment of inertia (see Eqn 6) and $\dot{\theta }$ is angular velocity. Rotational energy percentage was calculated as:

(3) $$KE_{R}\%=\frac{KE_{R}}{KE_{T}+KE_{R}}.$$

Vertical force was calculated as:

(4) $$F_{y}=m\;\ddot{y},$$

where $\ddot{y}$ is vertical acceleration.

The moments are calculated as:

(5) $$F_{y}\;b=I\ddot{\theta },$$

where $\ddot{\theta }$ is angular acceleration.

Finally, moment of inertia was defined as:

(6) $$I=\frac{1}{12}mL^{2},$$

where *L* is body length.

#### Hat mass calculation and equations

During take-off, spring potential energy (SPE) is converted into translational and rotational kinetic energy (Eqns 1 and 2) at a kinetic energy ratio of 98.7:1.3%, respectively, at take-off (Goode and Sutton, 2023):

(7) $$\frac{KE_{T}}{KE_{R}}=\frac{\frac{1}{2}\;\mathrm{mv}{\;}^{2}}{\frac{1}{2}\;\frac{1}{12}\;m\;\;L^{2}{\dot{\theta }}^{2}}\;=\;c,$$

where *c* is an unknown constant that cancels out of the equation. The force from the metathoracic legs (Fig. 1B) is applied to the body of the locust at an angle ϕ=45 deg from the ground, where only the vertical component of force (*F_y_*) (Eqn 4) produces rotational torque. Linear velocity (*v*) has a horizonal (*x*) and vertical (*y*) component, which can both be expressed in terms of vertical velocity ($\dot{y}$) (Eqn 8). Here, the mass (*m*) and coefficients of one half cancel out of the equation.

(8) $$\frac{\left(\;{\dot{y}}^{2}+{\left(\frac{\;\dot{y}}{\tan(\varphi )}\right)}^{2}\right)}{\frac{1}{12}\;L^{2}\;{\dot{\theta }}^{2}}=c\;.$$

Simplifying the kinetic energy ratio equation (Eqn 8) to make angular velocity ($\dot{\theta }$) the subject and differentiating both sides with respect to time (*t*), it is possible to calculate angular acceleration ($\ddot{\theta }$) in terms of vertical acceleration ($\ddot{y}$) and body length (*L*) (Eqn 9):

(9) $$\ddot{\theta }=\frac{\ddot{y}\;\sqrt{\left(1+{\left(\frac{1}{\tan(\varphi )}\right)}^{2}\right)}}{\sqrt{\frac{1}{12}}\;L\;\sqrt{c}}\;.$$

Substituting the force equation (Eqn 4) into the moment equation (Eqn 5) produces an equation for vertical acceleration ($\ddot{y}$), including the moment arm length (*b*) (Eqn 10):

(10) $$\ddot{y}=\frac{\frac{1}{12}\;L^{2}\ddot{\theta }\;\;}{b}\;.$$

Substituting the angular acceleration equation (Eqn 9) into the equation for translational acceleration (Eqn 10) produces an equation for the constant of the moment arm length (*b*); the distance between the centre of mass and the leg force vector, in terms of *L* (Eqns 11 and 12):

(11) $$b\;\frac{L}{b}\;=\;12\;\frac{\sqrt{\frac{1}{12}}\;\sqrt{c}}{\sqrt{\left(1+{\left(\frac{1}{\tan(\varphi )}\right)}^{2}\right)}},$$

(12) b=L/21.34.

Eqn 12 predicts that the length of *b* will be equal to 4.7% of the total body length, for any size locust.

Finally, it is possible to calculate the hypothetical hat mass (*m*_2_) required to cancel out rotational forces acting on the body using a simple balance beam model (Fig. 1B) to quantify the moment forces (Eqn 5) of the body mass (*m*_1_) (Eqns 13 and 14):

(13) $$m_{2}=\frac{m_{1}\;b\;}{L/2},$$

Taking the ratio between *m*_2_ and *m*_1_ (Eqn 14), we can calculate the hat mass percentage *h*% as:

(14) $$\frac{m_{2}}{m_{1}}=0.0937.$$

(15) $$h\%=100\;(m_{2}/m_{1}).$$

Thus, we predict that it will take a hat mass (*m*_2_) of *h*%=9.37% of the animal's body mass (*m*_1_) to move the centre of mass the distance of the moment arm length (*b*), such that the locust will produce no rotational kinetic energy and thus no angular rotation at take-off.

### Aim and hypotheses

In this study, we aimed to test whether it was possible to reduce rotational velocity by adding mass to the head of the locust without effecting the overall performance of the jump. From our hat mass calculation (Eqn 14), we predicted that a hat mass weighing 9.37% of the locust's body mass would be required to produce a jump with no angular rotation. Therefore, to test this, we measured locusts jumping under the five test conditions: *h*%=0%, 3.4%, 6.8%, 10.2% and 13.6%, with 0% being the control group, where no mass was added to the locusts.

In a latch mediated spring actuated system, the amount of energy at take-off is equal to the amount of energy stored in the spring prior to take-off. The energy in the spring is not affected by adding mass to the animal (Sutton et al., 2016; Sutton and Burrows, 2018) and thus, the translational kinetic energy$\;\left(\frac{1}{2}\;mv^{2}\right)$ of the locust at take-off should not vary with hat mass. Therefore, hypothesis 1 predicts that translational kinetic energy will not significantly vary with the increase of hat mass: KE_T_ (mJ) does not vary with *m*_2_.

However, conservation of translational kinetic energy predicts that linear velocity will proportionally decrease as the locust's total mass increases:

(16) $$\frac{1}{2}m_{i}v_{i}^{2}=\frac{1}{2}\;m_{f}v_{f}^{2},$$

where the subscripts i and f indicate initial and final values, respectively. Owing to the hat mass being proportional to the locust's body mass by *h*%, the conservation of energy equation can be written in terms of the locust's body mass (*m*_1_), where the initial mass (*m*_i_) is equal to *m*_1_, and the final mass (*m*_f_) is equal to *m*_1_(1+*h*%):

(17) $$\frac{1}{2}m_{1}v_{i}^{2}=\frac{1}{2}m_{1}(1+h\%)\;v_{f}^{2},$$

which simplifies to:

(18) $$\;v_{f}=v_{i}/\sqrt{\left(1+h_{\%}\;\right)}.\;$$

Using the mean linear velocity of the control group as the initial velocity (*v*_i=_1.57 m s^−1^), it is possible to calculate the final velocity (*v*_f_). The inverse square root relationship between velocity and hat mass (*m*_2_) can be linearly approximated (Table S1, Fig. S1) forming hypothesis 2, where velocity (m/s) varies with hat mass, via the equation:

(19) $$v=-0.0071m_{2}+1.57.$$

This predicts that when *h*%=13.6%, final velocity is equal to 1.47 m s^−1^, suggesting that the heaviest hat mass used in our study will reduce the linear velocity by less than 10%, from 1.57 to 1.45 m s^−1^.

Hypothesis 3, and the overall aim for this study, is that angular velocity will decrease as the hat mass increases. Eqn 14 calculates that when hat mass equals 9.37% of the body mass, the moments about the centre of mass will reach equilibrium, with zero net torque to generate angular rotation at take-off. Thus, when *m*_2_=0.0937*m*_1_, angular velocity $\dot{\theta }$ is 0 rad s^−1^. In the control group of this study, where there is no hat mass (*m*_2_=0), the average angular velocity *$\dot{\theta }$* is 12.99 rad s^−1^. From these two data points we can calculate the slope and intercept assuming a linear effect of hat mass on angular velocity:

(20) $$\dot{\theta }=-1.39\;m_{2}+12.99.$$

### Study organisms

Captive bred pre-adult moult, male and female, locusts (*Schistocerca gregaria* Forsskål 1775) ranging in body mass from 0.61 to 1.20 g were housed in colonies, in a terrarium at 24–30°C on a 12:12 h light:dark day cycle at the University of Lincoln Insectary. The locusts were fed bran-based insect feed (ProRep Bug Grub, UK) and polyacrylamide gel (ProRep Bug Gel, UK) *ad libitum*.

Locusts were selected from the colony at random, weighed using an analytical balance and measured from head to the tip of the abdomen with a ruler. From these measurements, masses, informally referred to as ‘hat masses’, were calculated and weighed out in increments of 3.4%, 6.8%, 10.2% and 13.6% of the body mass of each locust. The hat masses were made from 0.8 mm diameter lead free soldering wire, cut to length to provide the desired mass. The masses were then individually attached to the front of the locust's head using clear, nontoxic, cosmetic lacquer. This provided sufficient adhesion but also allowed the masses to be attached and removed without causing any damage to the locust. Applying the mass between the eyes to the centre of the anterior surface of the head caused no noticeable change to the locust's behaviour or willingness to jump.

### Ethics

All procedures were conducted in accordance with the United Kingdom Animal (Scientific Procedures) Act 1986 and were approved by the University of Lincoln ethical review committee.

### Filming and analysis

In total, nine locusts were filmed jumping under the five different weighted conditions. To control for the locust adjusting to the added mass, only five repetitions of each test condition were filmed per individual, resulting in a total of 225 jumps. The order in which the five test condition masses were added was randomised using a random number generator (Microsoft Excel), to reduce the likelihood of the locusts adjusting their behaviour to the increasing mass or showing exhaustion in later test conditions. However, previous work indicates that it is unlikely that locusts experience a decline in performance from repetitive jumping (Goode and Sutton, 2023).

**Fig. 2.**
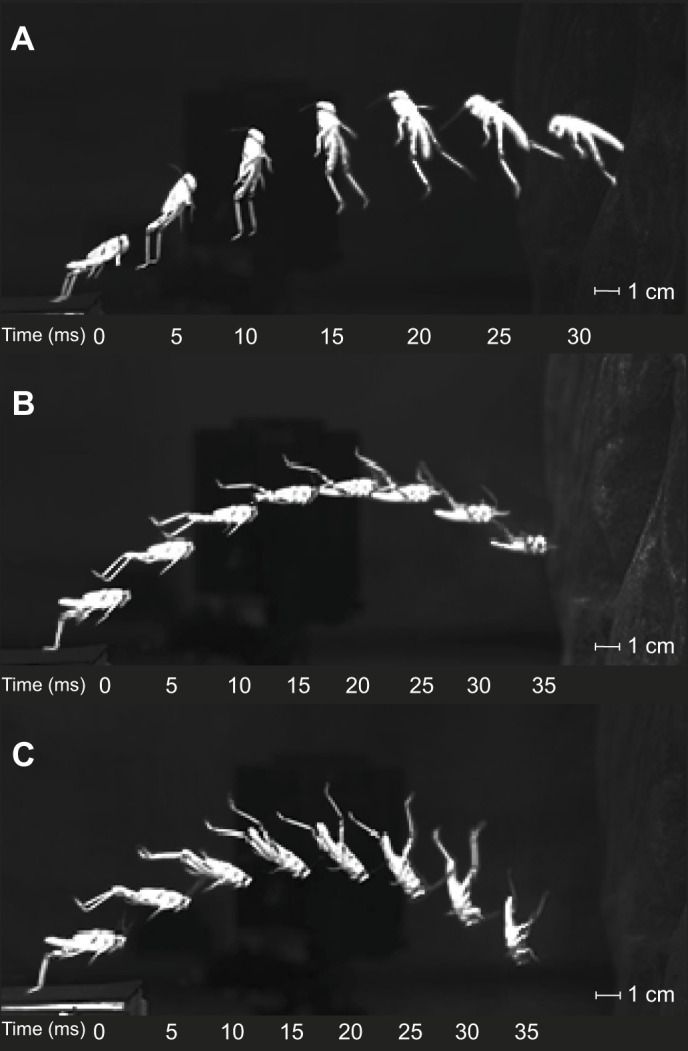
**High-speed movie stills of a locust jump.** (A) Locust jumping with typical head-up, tail-down rotation. Control test condition 0% of the body mass added to the head of the locust. (B) Jump with no angular rotation. Test condition: 6.8% of the body mass added to the head. (C) Jump with head-down, tail-up rotation. Test condition: 13.6% of the body mass added to the head. Representative data from animal 1 (ID: 260621), body mass: 0.72 g, body length: 35.00 mm.

Footage was recorded using a Photron FASTCAM Mini, from a lateral perspective, orthogonal to the direction of the jump (as shown in Fig. 2). Locusts were individually placed on a wooden podium (10.2×12.7×5.1 cm, height×width×depth), 30 cm away from the ‘target’, a horizontal bark-like textured terrarium wall. Locusts were encouraged to jump, from the podium to the wall, by quickly introducing a paintbrush into their visual field. Locusts were manually tracked using the Tracker video analysis and modelling tool (Open Source Physics, 2020). A scale bar was attached to the podium during filming to allow for the calibration of the tracking software, reducing the risk of lens distortion. The positions of the locust's head and centre of mass, determined as the point immediately behind the coxa joint of the metathoracic legs, were recorded as *x*,*y* coordinates at time points: take-off phase, *t*=0 ms, and 10 frames after take-off, *t*=10 ms. Take-off was determined as the point at which both of the metathoracic tarsi left the ground. The coordinates of the centre of mass and head were used to determine linear velocity (*v* in m s^−1^) and angular velocity ($\dot{\theta }$ in rad s^−1^), which, along with the moment of inertia (*I* in kg m^2^), were used to calculate translational kinetic energy (KE_T_ in mJ), rotational kinetic energy (KE_R_ in mJ) and the rotational energy percentage of the total energy [KE_R_/(KE_T_+KE_R_) in %] during the first 10 ms after take-off.

### Statistical analysis

All statistical models were computed within RStudio (R version 4.3.3, Posit Software, Boston, MA, USA), with a critical value of *P*≤0.05 used to determine statistical significance. Scaling coefficients were calculated using a fit linear mixed-effects model (lme4::lmer; Bates et al., 2015; Kuznetsova et al., 2017). This model allowed all 225 jumps, 45 for each of the five test conditions, to be used as individual data points, with repeat measures from individual animals being treated as a random factor: response∼factor+(1|random effect). The *R*^2^ values for these models were calculated using the MuMIn package (https://CRAN.R-project.org/package=MuMIn). This model was also used to calculate the analysis of covariance between angular velocity (response) and linear velocity (covariate), comparing the scaling coefficients, response∼factor×covariate+(1|random effect), and intercepts, response∼factor+covariate+(1|random effect), for each hat mass group (factor). *Post hoc* Tukey contrasts for pairwise comparisons of the means, for all of the lmer models, were calculated using a general linear hypotheses model (multcomp::glht; Hothorn et al., 2008). Finally, significance for the difference between the real and predicted slopes (data–prediction) for linear velocity and angular velocity were calculated using a linear regression test (Excel, Stats package).

## RESULTS

### Linear velocity

The average linear velocity for the control group was 1.57±0.29 m s^−1^ (Table S2), which is within the typical linear velocity range of 1.21±0.33 m s^−1^ (Goode and Sutton, 2023) to 3.1 m s^−1^ (Bennet-Clark, 1975) for a jumping locust found in previous studies.

**Fig. 3.**
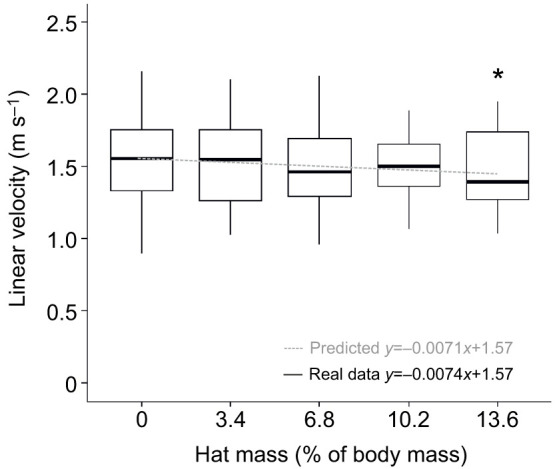
**Linear velocity (m s^−1^) of the locust's jump decreased as mass added to the locust's head increased.** Significant difference from the control 0% (*n*=45): *P<0.05.

Linear velocity at take-off (Fig. 3) had a significantly negative relationship with increasing hat mass: *v*=–0.0074*m*_2_+1.57 (*t*_215_=−3.21, *P*=0.002, *R*^2^=0.66; Table S3). However, the fixed effect of the hat mass alone accounted for very little of the difference in variation between the groups (marginal *R*^2^=0.02). Pairwise comparisons of the means indicated a statistically significant difference between the control group (0%) and the heaviest hat condition (13.8%) (*t*_218_=−3.02, *P*=0.02; Table S4). The slope of this relationship was not significantly different from our predicted slope, *y*=–0.0071*m*_2_+1.57 (*t*_224_=−0.07, *P*=0.95, *R*^2^=2.06×10^−5^; hypothesis 2).

The reduction in linear velocity owing to hat mass only became statistically significant after 13.8% of the body mass was added to the locust, whereby the mean linear velocity was only reduced by 0.09 m s^−1^, which was still within the normal linear velocity range for a jumping locust (Goode and Sutton, 2023). Therefore, we can assume that jump performance in this dataset is normal and will hereforth compare the rotational velocity kinematics and energetic results from all five test conditions without the need to adjust the data to account for any hat mass induced decrease in jump performance.

### Angular velocity

The average angular velocity for the control group was 12.99±5.31 rad s^−1^ (Table S2), which is within the typical angular rotation range of 1.75–51.08 rad s^−1^ previously recorded in locusts jumping (Goode and Sutton, 2023).

**Fig. 4.**
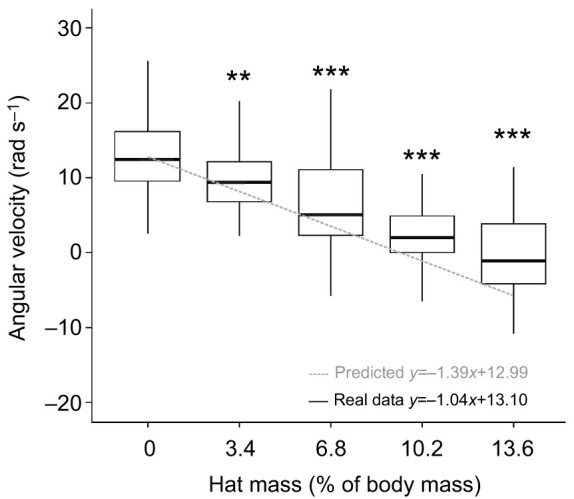
**Angular velocity (rad s^−1^) of the locust's jump decreased with increasing hat mass.** Significant difference from the control 0% (*n*=45): **P<0.01; ***P<0.001.

Angular velocity at take-off (Fig. 4) had a significantly negative relationship with increasing hat mass: $\dot{\theta }$=–1.04*m*_2_+13.10 (*t*_215_=−18.60, *P*<2e–16, *R*^2^=0.71; Table S3), causing locusts with greater hat mass to rotate with a more negative angular velocity, and thus become increasingly more head-down, tail-up oriented (Fig. 2C). The fixed effect of hat mass alone had a notable explanatory power for the variation between each group (marginal *R*^2^=0.45). Pairwise comparisons of the means indicated each test condition was significantly (*P*<0.05) different from the control ‘0%’ (Table S4). The slope for angular velocity was significantly different from the predicted slope of *y*=–1.39*m*_2_+12.99 (*t*_224=_4.69, *P*=4.76×10^−6^, *R*^2^=0.09) (hypothesis 3).

### Covariance of angular velocity and linear velocity

**Fig. 5.**
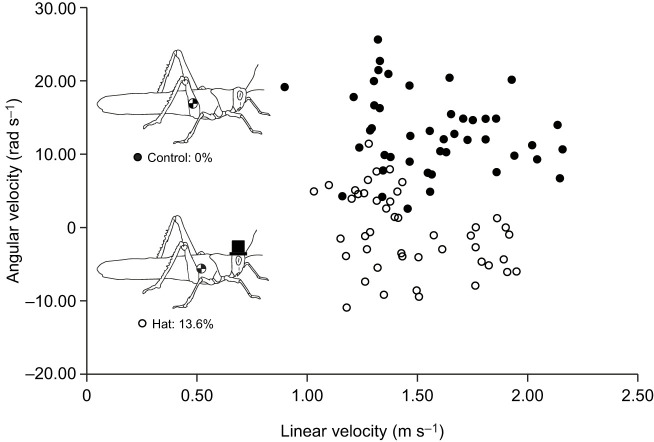
**Angular velocity (rad s^−1^) and linear velocity (m s^−1^) for a locust jump, comparing the control 0% (*n*=45) with the maximum hat mass 13.6% (*n*=45).** Correlations for the entire dataset, displaying the continuum between these two conditions, is shown in Table S6.

Angular velocity had a non-significant negative relationship with increasing linear velocity for the control group: $\dot{\theta }$=–1.19*v*+14.85 (*t*_224_=−0.53, *P*=0.60; Fig. 5; Table S6). The linear regression slope coefficients for test groups 3.4% (*t*_216_=2.63, *P*=0.009) and 6.8% (*t*_216_=2.86, *P*=0.005) were statistically different from the control; however, test groups 10.2% (*t*_216_=0.42, *P*=0.67) and 13.8% were not, showing an inconclusive relationship between linear and angular velocity as hat mass increased: $\dot{\theta }$=–5.15*v*+6.87 (*t*_216_=−1.34, *P*=0.18; Fig. 5; Table S6). The slope intercepts for all of the test groups were significantly different and increasingly more negative than the control, with the lowest intercept being hat mass 13.8% (*t*_217_=−15.71, *P*<2e-16; Table S6). This shows a disproportionate reduction in angular velocity than linear velocity as hat mass increased.

### Energetics

The average rotational kinetic energy (KE_R_ in mJ) for the control group was 1.17±1.26% (Table S2) of the total energy of the jump (KE_R_+ KE_T_). This was in keeping with a previous study where rotational kinetic energy was on average 1.3% of the total energy (translational kinetic energy+rotational kinetic energy+gravitational potential energy in mJ; Goode and Sutton, 2023).

**Fig. 6.**
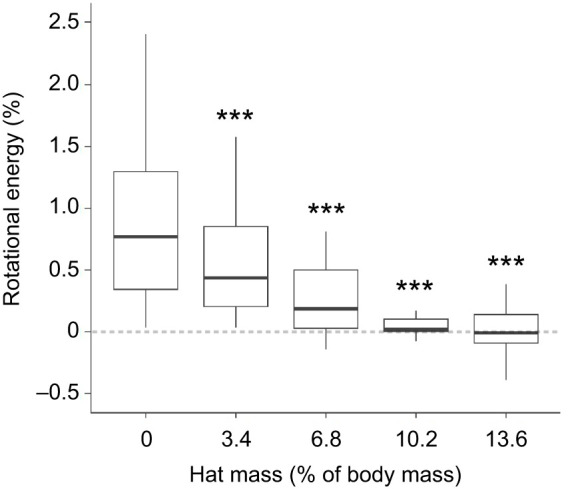
**Rotational energy, as a percentage of the total energy (translational and rotational kinetic energy) of the locust jump, decreased as hat mass increased.** Significant difference from the control 0% (*n*=45): ***P<0.001.

The percentage of rotational kinetic energy (Fig. 6) in the jump significantly decreased as hat mass increased (KE_R_=–0.09*m*_2_+1.05; *t*_215=_−11.06, *P*<2e-16, *R*^2^=0.57; Table S3), causing locusts with greater hat mass to have less rotational energy, as a percentage of their total energy. Pairwise analysis of the means indicated a statistically significant difference between all test groups from the control (Table S4).

### Analysis of consecutive jumps

**Fig. 7.**
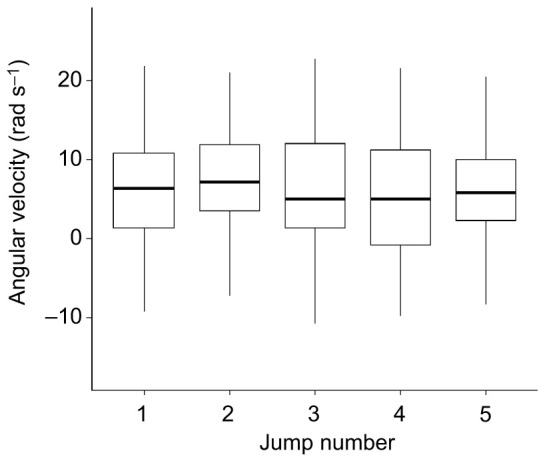
**Consecutive jump analysis of angular velocity.** In a sequence of five jumps performed under test conditions 0–13.6% (n=45), each consecutive jump was not statistically different from the first (*P*>0.05), showing little evidence for jump performance increasing over time.

To test whether the locusts were learning to compensate for their new mass distribution, we analysed the performance of each consecutive jump 1–5, independent of the test condition. Angular velocity had a non-significant negative relationship with increasing consecutive jump number: $\dot{\theta }$=−0.20×jump number+6.66 (Fig. 7). This slope was not significantly different from a slope of zero (*t*_215=_−0.66, *P*=0.51, *R*^2^=0.24; Table S2). The model's total explanatory power was weak, with the fixed effects of consecutive jump number alone accounting for very little of the variation seen within this model (marginal *R*^2^=0.001). Pairwise comparisons of the means did not find a statistically significant difference in angular velocity between any pair of jump number groups (Table S5). These results do not indicate any evidence of the locusts learning and adjusting their jump performance over time to account for the new mass added to their heads, to correct their artificially induced head-down angular velocity at take-off.

## DISCUSSION

To what extent is the angular rotation experienced by a locust during the take-off phase of a jump a by-product of the distribution of its mass? To investigate this, we measured the linear and angular velocity of *S. gregaria* jumping with incrementally increasing mass added to their heads. We observed that the addition of mass in such a manner significantly reduced the angular velocity (Fig. 4), whilst causing only a very small reduction in linear velocity (Fig. 3).

Our previous study (Goode and Sutton, 2023) demonstrated that the distribution of translational and rotational kinetic energy at the time of take-off was on average 98.7:1.3% of the total energy budget. Here, we effectively aimed to change this distribution ratio to 100:0%, purely by the addition of graded hat masses, thus altering the relative relationship between angular and linear velocity. In doing so, we found a significant and negative correlation between rotational energy percentage and hat mass, indicating that angular velocity decreased disproportionately more than linear velocity, as hat mass increased. Our calculations controlled for the mass dependency of rotational kinetic energy and angular velocity by considering the rotational energy as a percentage of the overall energy budget, and by calculating hat mass as a percentage of each animal's overall body mass, because smaller locusts typically rotate faster (Goode and Sutton, 2023). Therefore, this pattern reflects only the effect of hat mass on the geometric shift in mass distribution throughout the locust and is independent of overall body mass.

The data presented here therefore clearly show that it is possible to produce a jump with almost no rotation at both take-off and during the following 10 ms (Fig. 2B). A jump with this little body rotation is otherwise very rarely seen under standard experimental conditions, with locusts consistently showing the head-up, tail-down pitch seen in the control 0% hat condition (Fig. 2A) and in previous literature (Bennet-Clark, 1975; Sutton and Burrows, 2008; Cofer et al., 2010; Gvirsman et al., 2016; Goode and Sutton, 2023). For hat masses inclusive and upwards of 6.8% of body mass, negative (head-down, tail-up) angular rotations were recorded, most consistently in the 13.6% condition. In our previous studies, jumps with negative rotation have only occurred during ‘falling’ jumps, where the locust aims for the ground below the podium, and thus lacks the high energy kicking of the legs at take-off. Therefore, in this study, the graded decrease in angular velocity observed in response to increasing hat mass (Fig. 4) ultimately supports our original hypothesis, that body spin at take-off is a passive result of mass distribution. If pitching was instead the result of a physiological or self-correcting mechanism, then adding mass to the head would not have any effect on the direction of the rotation, as it would be expected that this control would be maintained to some extent when mass was added. The hat mass did increase the moment of inertia of the locust by up to 41% in the case of the heaviest hat. If we were to hypothetically assume that the centre of mass did not move, then the increase in moment of inertia would reduce the resultant angular rotation, but could not eliminate rotation entirely, nor reverse its direction. Therefore, the zeroing out of rotation, and rotation in the reverse direction (head-down, tail-up) seen in this study is most likely due to the movement of the centre of mass and not an increase in moment of inertia.

Although this study provides no empirical insight into the potential benefits of angular velocity during launch, the fact that zero rotation could be presumably achieved by a locust making minimal alterations to its body mass distribution implores us to hypothesise that jumping with less angular rotation is of no significant benefit to the animal. The rapidity of the take-off likely renders neural control of angular rotation – via the dorsal–longitudinal muscles – improbable, though regardless, this mechanism does not allow precise control and instead serves to dictate the general direction of rotation (Cofer et al., 2010). Because postural adjustments are already used to alter the jump's linear vector of movement (Santer et al., 2005; Sutton and Burrows, 2008; Cofer et al., 2010; Gvirsman et al., 2016), one might assume that if there was a pressing evolutionary benefit to jumping with less angular velocity, the alterations needed to control this precisely would be relatively easy to achieve and therefore would occur more frequently. Locust jumps tend to have angular velocities that are more variable and, in the case of smaller individuals, pitch faster than similarly sized bush crickets (*Mecopoda elongata*). Bush crickets jump with a muscle-actuated system and inhabit more complex, foliage-dense environments, which potentially act as a selection pressure for a jumping mechanism with a greater accuracy. A more stable angular velocity may allow them to jump with precise vertical trajectories to specific target substrates. The limitation here is that for smaller animals, the muscle actuated system results in a lower linear velocity than a spring actuated jumper (Goode et al., 2023). Because locusts inhabit more open environments, vertical precision is likely less important and might explain why they seem to lack a specific mechanism for controlling their angular velocity.

In our analysis of rotational kinetic energy, we found a correlation of *y*=–0.09*m*_2_+1.05, predicting that 0% rotational energy should be achieved when 11.66% of the body mass is added to the locust's head. The error between this prediction and that of our initial prediction of 9.37% (Eqn 14) can likely be attributed to the simplification of the locust's body shape and mass distribution in our uniform beam model. Similarly, the linear mixed effects regression model for angular velocity predicted that 0 rad s^−1^ would be reached at a hat mass of 12.59% of the body mass. These two values should not be inherently different, owing to rotational energy being the only part of the total energy budget contributing to the production of angular velocity. Therefore, this error is likely due to variation in jumping behaviour, energy input and energy distribution.

It is unsurprising that the increase in mass resulted in a small decrease in linear velocity, as the kinetic energy inputted into the jump remained constant throughout each condition. During growth, natural increases in mass are supplemented by a corresponding increase in metathoracic leg length and femoral muscle mass, so that the leg's power output increases proportionally with total body mass (Gabriel, 1985), and this concomitant growth is obviously lacking when mass is artificially added to the animal. Intra-individual differences likely account for the lack of a significant reduction in linear velocity for the smaller hat masses (3.4–10.2%), as locusts are known to store differing amounts of elastic energy in the leg prior to take-off (Bennet-Clark, 1975). Such variation might have masked changes in linear velocity when low amounts of mass were added. In addition to this, the difference in mean linear velocity between the 0% and 13.6% conditions was only 0.09 m s^−1^, considerably less than the intra-animal variation recorded in our previous study (Goode and Sutton, 2023). Because significant differences in linear velocity were only detectable once hat mass reached 13.6%, further research with a range of masses exceeding this value would help quantify this relationship more thoroughly. Our dataset once again indicated that linear and angular velocity appear to be approximately independent of one another, with relatively weak correlations being found between the two for all five hat conditions (Fig. 5). These correlations are weaker than in our previous work (Goode and Sutton, 2023, their fig. 5B, *R*^2^=0.31). This difference could be due to the fact that fewer consecutive jumps were recorded per individual in this study.

It has previously been shown that the jump performance of a locust, in terms of linear velocity, does not change over multiple consecutive jumps (Goode and Sutton, 2023). However, by adding a mass to the locust, we introduced a new factor that could potentially cause the locust to fatigue or alter their jumping behaviour to account for their new mass distribution, subsequently increasing jump performance over time. To control for this effect, the order in which each mass (0% to 13.6% body mass) was added to the locust was randomised. However, the locusts were jumped five times consecutively under each of the five mass conditions, during which the locusts could develop newly learnt compensatory jump behaviours. From the kinematic results (Fig. 4), we can see that angular velocity significantly decreases as hat mass increases. This suggests that the locusts do not immediately adjust their jump performance to account for the masses added, showing little evidence of immediate mediation from the vestibular system and proprioceptors. Analysis of consecutive jumps found that the angular velocity did not significantly differ from jump 1 to 5, indicating little evidence for learning over time as well as little evidence that animals fatigued.

To conclude, the results of this study strongly indicate that locusts are not controlling or adjusting their angular rotation during launch, nor within the first 10 ms after take-off. A very minimal shift in the distribution of their body mass is sufficient to entirely remove angular rotation from the jump. The calculated rotational energy percentages suggest that our simplified model of the locust, as a beam rotating around its centre, is sufficient to both accurately predict the forces acting upon it, and calculate the mass required to remove rotation from the jump of a locust of any size, to within a small degree of error.

## Supplementary Material

10.1242/jexbio.249846_sup1 Supplementary information
