# Supplementary material for: Control of high-speed jumps: removing rotation from the jumps of locusts (Schistocerca gregaria)
Source: J Exp Biol. 2026 Aug 7;229(15):jeb249846. doi: 10.1242/jeb.249846 (PMC13480952; doi:10.1242/jeb.249846)
Supplement: Supplementary file 1 [file jexbio-229-249846-s1.pdf]

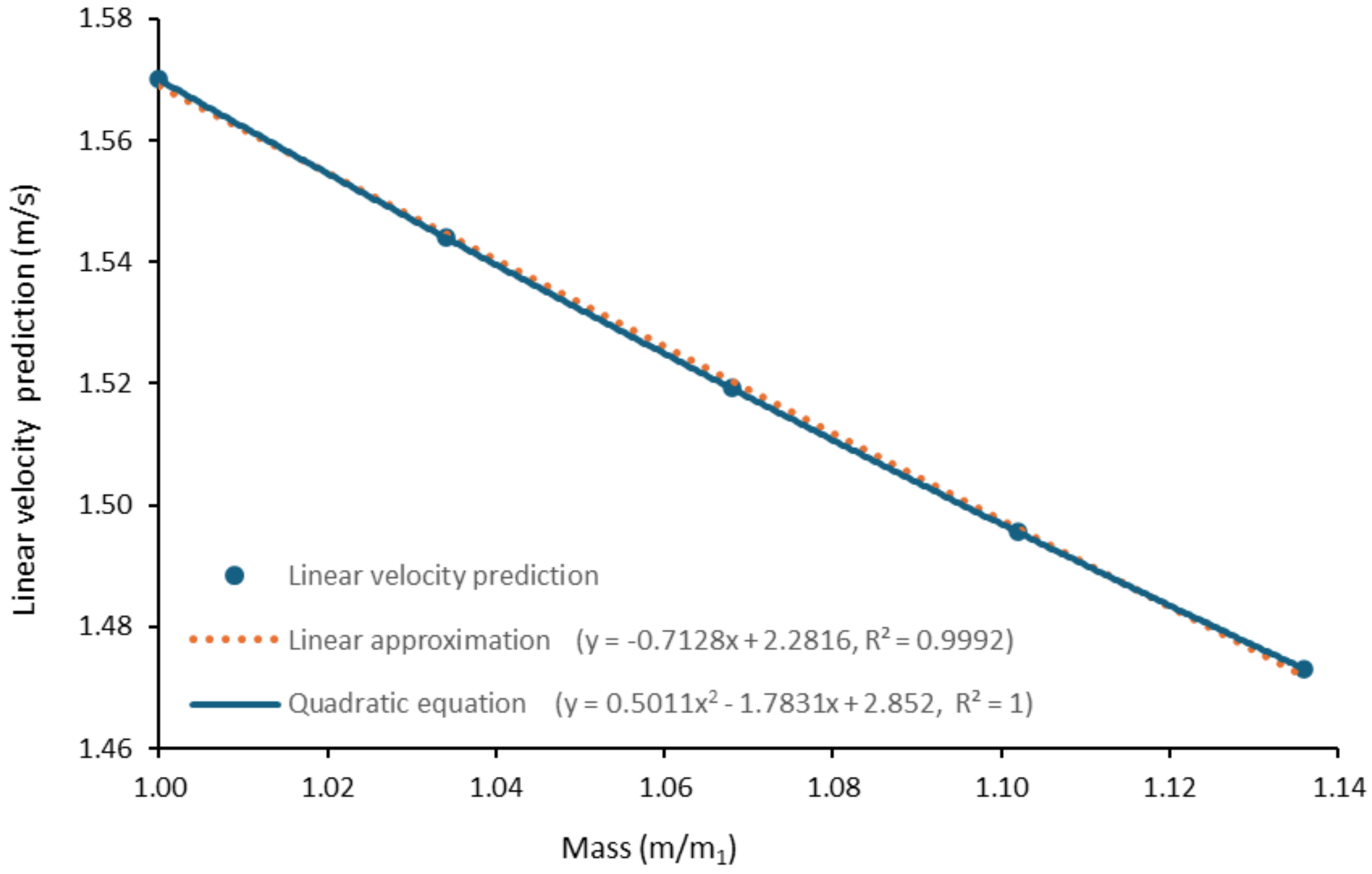

Fig. S1. Linear velocity prediction for hypothesis two, calculated from the conservation of energy equation. Comparison of the quadratic equation and linear approximation equation, fit by the method of least squares. This cur

Table S1. Linear velocity calculated from the quadratic equation and linear equation trendlines in Figure S1. The linear approximation of linear velocity is identical to three significant digits to the quadric equation.

Mass		***Linear velocity (m/s)***	
h%	*m/m1*	*Quadratic Equation (y = 0.50x2 -1.78x +2.85)*	*Linear approximation (y = - 0.73x + 2.28)*
0	*1.000*	*1.570*	*1.569*
3.4	*1.034*	*1.544*	*1.545*
6.8	*1.068*	*1.519*	*1.520*
10.2	*1.102*	*1.496*	*1.496*
13.6	*1.136*	*1.473*	*1.472*

Journal of Experimental Biology • Supplementary information

Table S2. Means (M) and standard deviations (SD).

Variable	*Factor*	*M*	*SD*
Linear velocity (m/s)	*Hat 0%*	*1.57*	*0.29*
	Hat 3.4%	*1.54*	*0.30*
	Hat 6.8%	*1.52*	*0.29*
	Hat 10.2%	*1.51*	*0.19*
	Hat 13.6%	*1.46*	*0.25*
Angular velocity (rad/s)	*Hat 0%*	*12.99*	*5.31*
	Hat 3.4%	*9.82*	*4.63*
	Hat 6.8%	*6.24*	*5.98*
	Hat 10.2%	*1.88*	*5.44*
	Hat 13.6%	*-0.66*	*5.29*
Rotational energy (%)	*Hat 0%*	*1.17*	*2.16*
	Hat 3.4%	*0.69*	*0.79*
	Hat 6.8%	*0.35*	*0.42*
	Hat 10.2%	*0.10*	*0.34*
	Hat 13.6%	*0.0051*	*0.29*
Angular velocity (rad/s)	*Jump 1*	*6.34*	*7.84*
	Jump 2	*6.49*	*7.44*
	Jump 3	*5.92*	*6.74*
	Jump 4	*5.89*	*7.38*
	Jump 5	*5.64*	*7.09*

Table S3. Linear mixed effects regression model results for kinematic and energetic data. Formula: lmer (y ~ x + (1| Animal)), where animal is a random effect.

Variables (y ~ x)	*Intercept*	*Coefficient*	*SE*	*df*	*t value*	*P value*	*R^2c*	*R^2m*
LV ~ Hat	*1.57*	*-0.007*	*0.002*	*215*	*-3.21*	*0.0015*	*0.66*	*0.02*
AV ~ Hat	*13.10*	*-1.04*	*0.056*	*215*	*-18.60*	*< 2e -16*	*0.71*	*0.45*
RE ~ Hat	*1.05*	*-0.09*	*0.008*	*215*	*-11.06*	*< 2e -16*	*0.57*	*0.24*
AV ~ Jump	*6.66*	*-0.20*	*0.306*	*215*	*-0.66*	*0.51*	*0.51*	*0.24*

Journal of Experimental Biology • Supplementary information

Table S4. Tukey Contrasts results for pairwise analysis of the means. Independent variable (x) of "hat mass" labelled as the following: 0% = h1, 3.4% = h2, 6.8% = h3, 10.2% = h4, 13.6% =

Variable	*x2 – x1*	*Estimate*	*Std. error*	*Z value*	*P value*
**Linear velocity**	h2 – h1	*-0.026*	*0.035*	*-0.73*	*0.95*
	h3 – h1	*-0.052*	*0.035*	*-1.49*	*0.57*
	h4 – h1	*-0.064*	*0.035*	*-1.83*	*0.36*
	h5 – h1	*-0.106*	*0.035*	*-3.02*	*0.02*
	h3 – h2	*-0.027*	*0.035*	*-0.76*	*0.94*
	h4 – h2	*-0.039*	*0.035*	*-1.10*	*0.81*
	h5 – h2	*-0.080*	*0.035*	*-2.29*	*0.15*
	h4 – h3	*-0.012*	*0.035*	*-0.35*	*1.00*
	h5 – h3	*-0.054*	*0.035*	*-0.15*	*0.54*
	h5 – h4	*-0.042*	*0.035*	*-1.19*	*0.76*
**Angular velocity**	h2 – h1	*-3.16*	*0.850*	*-3.72*	*0.002*
	h3 – h1	*-6.74*	*0.850*	*-7.94*	*<1e -04*
	h4 – h1	*-11.10*	*0.850*	*-13.08*	*<1e -04*
	h5 – h1	*-13.64*	*0.850*	*-16.06*	*<1e -04*
	h3 – h2	*-3.58*	*0.850*	*-4.22*	*0.0002*
	h4 – h2	*-7.95*	*0.850*	*-9.36*	*<1e -04*
	h5 – h2	*-10.48*	*0.850*	*-12.34*	*<1e -04*
	h4 – h3	*-4.36*	*0.850*	*-5.14*	*<1e -04*
	h5 – h3	*-6.90*	*0.850*	*-8.12*	*<1e -04*
	h5 – h4	*-2.54*	*0.850*	*-2.99*	*0.024*
**Rotational Energy**	h2 – h1	*-0.48*	*0.117*	*- 4.13*	*<0.001*
	h3 – h1	*-0.82*	*0.117*	*-7.02*	*<0.001*
	h4 – h1	*-1.07*	*0.117*	*-9.17*	*<0.001*
	h5 – h1	*-1.17*	*0.117*	*-9.98*	*<0.001*
	h3 – h2	*-0.34*	*0.117*	*-2.89*	*0.032*
	h4 – h2	*-0.59*	*0.117*	*-5.04*	*<0.001*
	h5 – h2	*-0.68*	*0.117*	*-5.85*	*<0.001*
	h4 – h3	*-0.25*	*0.117*	*-2.15*	*0.198*
	h5 – h3	*-0.35*	*0.117*	*-2.96*	*0.026*
	h5 – h4	*-0.09*	*0.117*	*-0.81*	*0.928*

Journal of Experimental Biology • Supplementary information

Table S5. Tukey Contrasts results for pairwise analysis of the means. Independent variable (x) of "Jump number" labelled J1 – J5.

Variable	*x2 – x1*	*Estimate*	*Std. error*	*Z value*	*P value*
**Angular velocity (rad/s)**	J2 – J1	*0.15*	*1.38*	*0.12*	*1.00*
	J3 – J1	*-0.42*	*1.38*	*-0.30*	*1.00*
	J4 – J1	*-0.45*	*1.38*	*-0.33*	*1.00*
	J5 – J1	*-0.70*	*1.38*	*-0.51*	*0.99*
	J3 – J2	*-0.57*	*1.38*	*-0.41*	*0.99*
	J4 – J2	*-0.60*	*1.38*	*-0.44*	*0.99*
	J5 – J2	*-0.85*	*1.38*	*-0.62*	*0.97*
	J4 – J3	*-0.36*	*1.38*	*-0.03*	*1.00*
	J5 – J3	*-0.28*	*1.38*	*-0.21*	*1.00*
	J5 – J4	*-0.25*	*1.38*	*-0.18*	*1.00*

Journal of Experimental Biology • Supplementary information

Table S6. Covariance between Angular velocity and Linear velocity.

	Variable	*Estimate*	*Std.error*	*df*	*t value*	*P value*
**Covariance– Scaling coefficient**	Intercept	14.85	3.78	184.69	3.93	0.0001
	LV	-1.19	2.26	224.30	-0.53	0.60
	Hat 3.4%	-14.19	4.29	216.20	-3.31	0.001
	Hat 6.8%	-18.88	4.36	216.44	-4.33	2.3e -05
	Hat 10.2%	-13.49	5.57	216.77	-2.42	0.02
	Hat 13.8%	-7.98	4.55	216.12	-1.75	0.08
	LV: Hat 3.4%	7.13	2.71	216.22	2.63	0.009
	LV: Hat 6.8%	7.96	2.78	216.43	2.86	0.005
	LV: Hat 10.2%	1.54	3.63	216.96	0.42	0.67
	LV: Hat 13.8%	-3.96	2.97	216.20	-1.34	0.18
**Covariance – Intercept**	Intercept	9.46	2.82	103.40	3.35	0.001
	LV	2.25	1.58	218.42	1.42	0.16
	Hat 3.4%	-3.10	0.84	215.99	-3.71	0.0003
	Hat 6.8%	-6.63	0.84	216.22	-7.88	1.6e -13
	Hat 10.2%	-10.96	0.84	216.37	-13.01	< 2e -16
	Hat 13.8%	-13.41	0.85	217.11	-15.71	< 2e -16

Journal of Experimental Biology • Supplementary information
